# The Network of Interactions Between Classical Swine Fever Virus Nonstructural Protein p7 and Host Proteins

**DOI:** 10.3389/fmicb.2020.597893

**Published:** 2020-11-30

**Authors:** Jindai Fan, Mengru Zhang, Chenchen Liu, Mengjiao Zhu, Zilin Zhang, Keke Wu, Zhaoyao Li, Wenhui Li, Shuangqi Fan, Chunmei Ju, Lin Yi, Hongxing Ding, Mingqiu Zhao, Jinding Chen

**Affiliations:** ^1^Department of Microbiology and Immunology, College of Veterinary Medicine, South China Agricultural University, Guangzhou, China; ^2^Guangdong Laboratory for Lingnan Modern Agriculture, College of Veterinary Medicine, South China Agricultural University, Guangzhou, China; ^3^Key Laboratory of Zoonosis Prevention and Control of Guangdong Province, Guangzhou, China

**Keywords:** classical swine fever virus, p7, protein interaction, interaction network, ubiquitin-proteasome system

## Abstract

Classical swine fever (CSF) is a highly contagious viral disease causing severe economic losses to the swine industry. As viroporins of viruses modulate the cellular ion balance and then take over the cellular machinery, blocking the activity of viroporin or developing viroporin-defective attenuated vaccines offers new approaches to treat or prevent viral infection. Non-structural protein p7 of CSF virus (CSFV) is a viroporin, which was highly involved in CSFV virulence. Deciphering the interaction between p7 and host proteins will aid our understanding of the mechanism of p7-cellular protein interaction affecting CSFV replication. In the present study, seven host cellular proteins including microtubule-associated protein RP/EB family member 1 (MAPRE1), voltage-dependent anion channel 1 (VDAC1), proteasome maturation protein (POMP), protein inhibitor of activated STAT 1 (PIAS1), gametogenetin binding protein 2 (GGNBP2), COP9 signalosome subunit 2 (COPS2), and contactin 1 (CNTN1) were identified as the potential interactive cellular proteins of CSFV p7 by using yeast two-hybrid (Y2H) screening. Plus, the interaction of CSFV p7 with MAPRE1 and VDAC1 was further evaluated by co-immunoprecipitation and GST-pulldown assay. Besides, the p7-cellular protein interaction network was constructed based on these seven host cellular proteins and the STRING database. Enrichment analysis of GO and KEGG indicated that many host proteins in the p7-cellular protein interaction network were mainly related to the ubiquitin-proteasome system, cGMP-PKG signaling pathway, calcium signaling pathway, and JAK-STAT pathway. Overall, this study identified potential interactive cellular proteins of CSFV p7, constructed the p7-cellular protein interaction network, and predicted the potential pathways involved in the interaction between CSFV p7 and host cells.

## Introduction

Classical swine fever (CSF), caused by CSF virus (CSFV), is a severe viral disease causing severe economic losses to the swine industry (Paton and Greiser-Wilke, 2003). Although a series of vaccination strategies have been developed to curb the outbreak events, CSF is still posing an ongoing threat to the swine industry. Early studies reported that the emergence of CSFV strains with moderate or attenuated virulence could result in persistent recessive infection and immunosuppression in pigs, making it more intractable to suppress and control CSF (Edwards et al., 2000; Moennig, 2000). Even though numerous studies related to the mechanism of CSFV replication have been performed, the pathogenesis of CSF is still poorly understood. Thus, the persistent research on the pathogenesis of CSF will be beneficial to the development of new vaccines or therapies for effectively controlling CSF.

CSFV, belonging to the Pestivirus genus within the Flaviviridae family, is a small, enveloped virus with a 12.3 kb single positive-sense RNA genome. Its genome consists of a single large open reading frame (ORF) flanked by a 5’-untranslated region (5’-UTR) and a 3’-UTR. The ORF encodes a polypeptide precursor, which could be cleaved into twelve mature proteins, including four structural proteins (C, E^*rns*^, E1, and E2) and eight non-structural proteins (N^*pro*^, p7, NS2, NS3, NS4A, NS4B, NS5A, and NS5B) (Thiel et al., 1991).

CSFV p7, a non-structural protein of approximately 6–7 kDa, could be easily degraded by the proteasome and was thus considered to be a short-lived protein (Lin et al., 2014). As a class II viroporin, CSFV p7 has been proven to be the small hydrophobic transmembrane protein. It could possess a short area of charged residues flanked by stretches of hydrophobic amino acids. These charged residues were predicted to constitute a cytosolic loop and two transmembrane helices, respectively (Gladue et al., 2012). Moreover, a previous study claimed that the C-terminal transmembrane helix of CSFV p7 processed the pH-dependent pore-forming activity (Gladue et al., 2012). Specifically, two sequence determinants including the C-terminal transmembrane helix (comprised by residues 39–67) and the preceding polar loop (residues 33–38) were confirmed to participate in the regulation of the pore-forming activity (Largo et al., 2014). Besides, it was showed that p7 could interact with host protein CAMLG, an integral endoplasmic reticulum (ER) transmembrane protein involved in intracellular calcium release regulation and signal response generation, and could efficiently mediate calcium permeability in the ER (Gladue et al., 2018). Recently, through the reverse genetics approaches, some scholars found that CSFV p7 was involved in CSFV virulence (Gladue et al., 2012) and was indispensable for virus production in cell cultures and could induce the secretion of IL-1β (Lin et al., 2014).

The yeast two-hybrid (Y2H) system is an effective way to examine the interaction of proteins and is frequently used to search for unknown interactive cellular proteins (Kang et al., 2012; Zhang et al., 2014). Screening of genome-wide cDNA libraries has become an efficient way to systematically discover cellular proteins that might interact with the viral protein. Then, the functional protein-protein interaction (PPI) analysis is an important approach to revealing the potential pathogenic mechanism of viral infection based on the interactions of proteins. In the context, to better characterize the role of p7 on the viral infection, we introduced the Y2H screening system, PPI network as well as GO enrichment and KEGG pathway analysis into this study and then predicted the potential pathways involved in the interaction between CSFV p7 and host proteins.

## Materials and Methods

### Cells and Antibodies

Human embryonic kidney (HEK293T) cells were cultured in Dulbecco’s modified Eagle’s medium (DMEM) complemented with 10% (v/v) fetal bovine serum (FBS) at 37^*o*^C in a humidified incubator with 5% CO_2_.

Primary antibodies employed in this study included mouse monoclonal anti-flag (AF519, Beyotime, Shanghai, China), mouse monoclonal anti-GFP (AG281, Beyotime), and rabbit monoclonal anti-GST (AF2299, Beyotime). Secondary antibodies included horseradish peroxidase (HRP)-conjugated goat anti-mouse IgG (H + L) (A0216, Beyotime), and HRP-conjugated goat anti-rabbit IgG (H + L) (A0208, Beyotime).

### Construction of Recombinant Plasmids

To construct the recombinant expression plasmids, including pGBKT7-p7 (BD-p7), pEGFP-p7, pET-N-GST-p7, p3 × Flag-MAPRE1, and p3 × Flag-VDAC1, genes *MAPRE1*, *VDAC1*, and CSFV *p7* were amplified by RT-PCR and cloned into the respective vectors. The primers used for amplifying *MAPRE1*, *VDAC1*, and CSFV *p7* were shown in Table 1.

**TABLE 1:** Primers used in this study.

Primer	Sequence (5′-3′)
pGBKT7-p7-F	CATGGAGGCCGAATTCCCATTGGGTCAGGGTGAGG
pGBKT7-p7-R	GCAGGTCGACGGATCCTCCACCCTTGGCAACCCCG
	CTAA
pET-N-GST-p7-F	CCCAAGCTTCCATTGGGTCAGGGTGAGG
pET-N-GST-p7-R	CCGCTCGAGTCCACCCTTGGCAACCCCGCTAA
pEGFP-p7-F	CCCAAGCTTCCATTGGGTCAGGGTGAGG
pEGFP-p7-R	CGGGATCCCGTCCACCCTTGGCAACCCCGCTAA
p3 × Flag-MAPRE1-F	CGGAATTCAATGGCAGTGAATGTGTACTCA
p3 × Flag-MAPRE1-R	GAAGATCTTTAATACTCTTCTTGCTCCTC
p3 × Flag-VDAC1-F	ATAAGAATGCGGCCGCGATGGCTGTGCCACCCACG
	TAT
p3 × Flag-VDAC1-R	GAAGATCTTTATGCTTGAAACTCCAGTCC

### Yeast Two-Hybrid (Y2H) Screening

A cDNA library of porcine peripheral blood mononuclear cell (PBMC) for Y2H screening was constructed in our previous work (Deng et al., 2019) and stored in our laboratory. After we confirmed that bait pGBKT7-p7 has no self-activating activity and is not toxic to yeast strain Y2HGold, pGBKT7-p7 was transformed into Y2HGold strain and screened against with the PBMC cDNA library using the Matchmaker Yeast Two-Hybrid System (630489, Clontech, United States). Transformants were selected on synthetically defined medium lacking His, Leu, Trp, and Ade (SD/-4) for 3–6 days at 30°C. Colonies were transferred to SD/-4 medium containing X-α-Gal and aureobasidin A (Aba) (SD/-4/X-α-Gal/Aba) for 3–5 days at 30°C. Plasmids from blue colonies were rescued, and targeted insertions were sequenced. The NCBI BLAST program was used to identify the host cellular proteins. The functional analysis of host cellular proteins was based on the UniProt database. To confirm the interaction between CSFV p7 and host cellular proteins, the bait and prey plasmids were co-transformed into the Y2HGold strain. Murine p53 and SV40 large T-antigen were co-transformed into the Y2HGold as positive control. Lamin C, which did not interact with SV40 large T-antigen, was regarded as a negative control.

### Co-immunoprecipitation (co-IP) Assays

HEK 293T cells were co-transfected with pEGFP-p7 and p3 × Flag plasmids using Lipofectamine^TM^ 3000 Transfection Reagent (L3000008, Thermo Fisher, United States) according to the manufacturer’s instructions. The cells were harvested at 24 h post-transfection, washed three times with cold PBS (pH 7.4), and lysed with lysis buffer for western and IP (P0013, Beyotime) containing 1mM PMSF (ST506, Beyotime) at 4°C for 30 min. Clarified extracts were incubated with an anti-GFP monoclonal antibody (mAb) for 12 h, incubated with 30 μL Protein A/G beads for 1 h, washed five times with IP wash buffer, boiled in loading buffer, and subjected to SDS-PAGE followed by immunoblotting with anti-GFP and anti-Flag mAbs.

### Glutathione S-Transferase (GST)-Pulldown Assays

The recombinant plasmid pET-N-GST-p7 was transformed into *E. coli* BL21 (DE3) competent cells. The expression of GST or GST-p7 protein was induced by the addition of 1 mM IPTG (ST098, Beyotime). The bacterial cells were harvested and resuspended in cold PBS containing 1 mM PMSF, followed by mild sonication. GST or GST-p7 protein was purified with BeyoGold^TM^ GST-tag Purification Resin (P2250, Beyotime) according to the manufacturer’s instructions and then incubated with 200 μL of the lysates of HEK293T cells transfected with p3 × Flag plasmids for 2 h at 4°C. The resin was further washed five times with cold PBS, followed by protein detection by SDS-PAGE and immunoblotting.

### Western Blotting

Protein samples were prepared as above and the concentration of protein was determined using a BCA protein assay kit (P0012, Beyotime). Protein samples were boiled in a 5 × SDS-PAGE loading buffer for 5 min. An equal amount of proteins was separated by SDS-PAGE and then transferred to polyvinylidene membranes (IPVH00010, Millipore, Germany). Membranes were blocked with 5% skim milk dissolved in PBS plus Tween-20 at 37°C for 1 h. Next, the membranes were incubated with primary antibodies at 4°C overnight and the corresponding secondary antibodies at 37°C for 1 h, at appropriate dilutions. The ECL Plus kit (P0018S, Beyotime) and a chemiluminescence imaging system (Fine-do X6; Tanon) were utilized to visualize the protein bands.

### Construction of the p7-Cellular Protein Interaction Network

The PPI network was employed to reveal functional relationships and interactions among proteins using the Search Tool for the Retrieval of Interacting Genes (STRING) (version 11.0) database^1^ (Szklarczyk et al., 2011) and Cytoscape v3.7.2 software. STRING database supplies information related to proven and predicted interactions among huge numbers of proteins and Cytoscape v3.7.2 software is a powerful bioinformatics platform for visualizing and analyzing the PPI network. In the present study, the potential binding partners of the CSFV p7 (Table 2) that were identified by Y2H assay were inputted into the STRING database, while the specie was set to “*Sus scrofa*” and other parameters were the default settings. The total list of proteins directly and indirectly related to potential binding partners of the CSFV p7 based on protein-protein interactions was obtained from the STRING database. Along with the experimentally confirmed interactions, those predicted from gene neighborhood, gene fusions, gene co-occurrence, text mining, co-expression, protein homology, and curated databases were also adopted for network construction and analysis. As an interaction score > 0.7 was considered significant, protein interactions from the STRING database with a combined score > 0.7 were selected for subsequent PPI network construction. According to the results of Y2H screening, the interaction score of p7 with seven identified host cellular proteins (Table 2) was set to 0.999. Based on the experimental results screened by the Y2H system and the correlation between the proteins in the STRING database, the p7-cellular protein interaction network was built using the Cytoscape v3.7.2 software. In the network, p7 protein is a core protein, nodes were implemented to represent the cellular proteins, and the interactions between proteins were expressed by straight lines between the nodes.

**TABLE 2:** Potential binding partners of the CSFV p7 protein.

Gene	Protein	NCBI accession	Function
MAPRE1	microtubule-associated protein RP/EB family member 1	NM_001044613.1	Involved in protein localization to microtubule plus-end, regulation of microtubule polymerization or depolymerization, spindle assembly, and cell migration.
VDAC1	voltage-dependent anion channel 1	XM_005652949.3	Involved in inorganic anion transport, apoptotic process, regulation of reactive oxygen species metabolic process, epithelial cell differentiation, behavioral fear response, synaptic transmission, and regulation of autophagy of mitochondrion.
POMP	proteasome maturation protein	XM_030882596.1	Involved in proteasome assembly.
PIAS1	protein inhibitor of activated STAT 1	XM_003121749.6	Involved in protein sumoylation, regulation of transcription by RNA polymerase II, protein-DNA complex assembly, regulation of cell proliferation and differentiation, and regulation of the apoptotic process.
COPS2	COP9 signalosome subunit 2	XM_021072083.1	Involved in regulation of nucleic acid-templated transcription, neuron differentiation, protein phosphorylation, and protein deneddylation.
CNTN1	contactin 1	XM_021092536.1	Involved in nervous system development, regulation of sodium ion transport, and regulation of gene expression.
GGNBP2	gametogenetin binding protein 2	XM_021067333.1	Involved in regulation of cell proliferation and differentiation, labyrinthine layer blood vessel development, regulation of gene expression, regulation of phosphorylation of STAT protein, and regulation of protein tyrosine kinase activity.

### Gene Ontology Term and KEGG Pathway Enrichment Analysis

Gene Ontology (GO) term enrichment analysis and Kyoto Encyclopedia of Genes and Genomes (KEGG) pathway analysis were carried out using the online tool Database for Annotation, Visualization and Integration Discovery tool (DAVID, https://david.ncifcrf.gov/) (Huang da et al., 2009a, b). GO enrichment includes 3 terms: molecular function (MF), biological process (BP), and cellular component (CC). A *p* < 0.05 was regarded as statistically significant for the correlations. The results of GO and KEGG pathways enrichment were visually analyzed using the RStudio, Version 1.3.1073 (RStudio Inc., Boston, MA, United States).

## Results

### Y2H Screening

**FIGURE 1:**
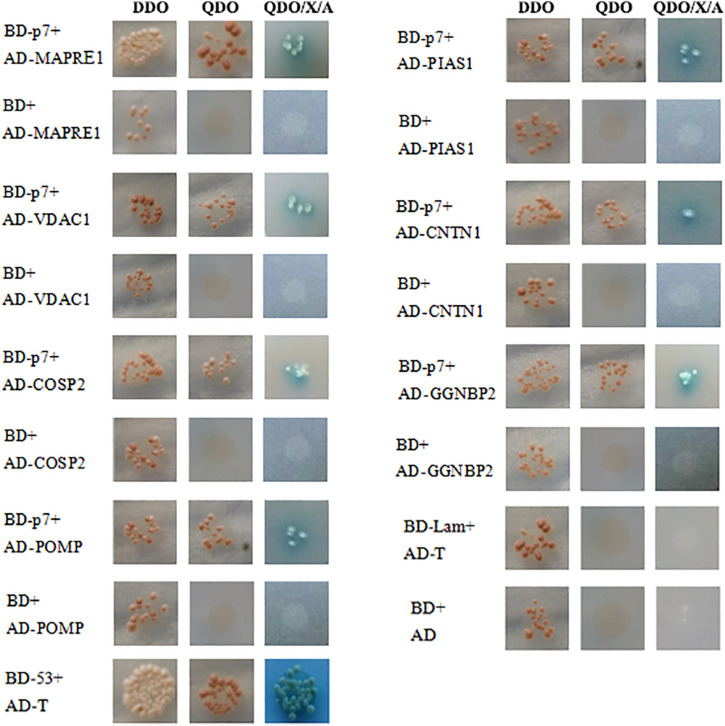
Identification of the interaction of CSFV p7 with host cellular proteins by the Yeast two-hybrid assay. To confirm the interaction between CSFV p7 and host cellular protein, the yeast strain Y2HGold was co-transformed with pGBKT7-p7 (BD-p7)/pGADT7-cellular protein (AD-cellular protein), pGBKT7 (BD)/AD-cellular protein (negative control), pGBKT7-p53 (BD-53)/pGADT7-T (AD-T) (positive control), pGBKT7-Lamin (BD-Lamin)/AD-T (negative control), and BD/pGADT7 (AD) (negative control). Host cellular proteins included microtubule-associated protein RP/EB family member 1 (MAPRE1), voltage-dependent anion channel 1 (VDAC1), proteasome maturation protein (POMP), protein inhibitor of activated STAT 1 (PIAS1), gametogenetin binding protein 2 (GGNBP2), COP9 signalosome subunit 2 (COPS2), and contactin 1 (CNTN1). Yeast cells co-transformed with AD-cellular protein and BD-p7 could grow on three kinds of synthetically defined media and form blue colonies on QDO/X/A medium, which indicated that the host cellular protein interacted with CSFV p7 protein in yeast cells.

The recombinant bait plasmid pGBKT7-p7 was screened against the PBMC cDNA library, and seven proteins were identified as potential binding partners of the CSFV p7 and their potential functions were listed in Table 2 based on the UniProt database. The identified proteins included microtubule-associated protein RP/EB family member 1 (MAPRE1), voltage-dependent anion channel 1 (VDAC1), proteasome maturation protein (POMP), protein inhibitor of activated STAT 1 (PIAS1), gametogenetin binding protein 2 (GGNBP2), COP9 signalosome subunit 2 (COPS2), and contactin 1 (CNTN1). As yeast cells co-transformed with pGADT7-cellular protein and pGBKT7-p7 could grow on three kinds of synthetically defined media and form blue colonies on QDO/X/A medium (Figure 1), it implied that the host cellular protein interacted with CSFV p7 in yeast cells.

### CSFV p7 Interacts With MAPRE1 and VDAC1, Respectively

**FIGURE 2:**
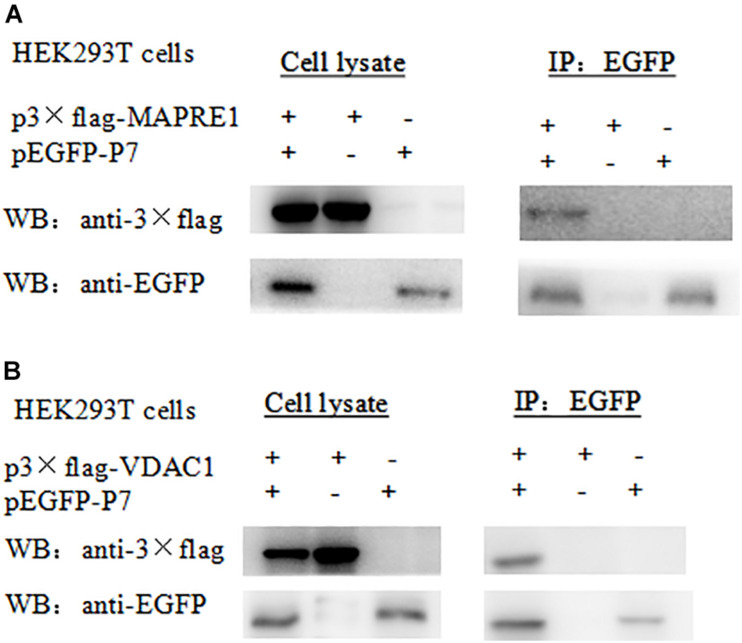
Identification of the interaction of CSFV p7 with MAPRE1 **(A)** and VDAC1 **(B)** by co-IP assay, respectively. Co-immunoprecipitation assay demonstrated that CSFV p7 bonded MAPRE1 and VDAC1 in HEK 293T cells, respectively. HEK 293T cells were co-transfected with EGFP-p7 and p3 × Flag plasmids and harvested at 24 h post-transfection. Cell lysates were immunoprecipitated with antibodies against EGFP or antibody against p3 × Flag followed by Western blot analysis.

For the Co-IP assay, HEK 293T cells were transformed with pEGFP-p7 and p3 × Flag plasmids, and cell lysates were immunoprecipitated with antibodies against EGFP followed by western blot analysis. Co-immunoprecipitation assay demonstrated that CSFV p7 could interact with MAPRE1 and VDAC1 in transfected HEK 293T cells, respectively (Figure 2).

**FIGURE 3:**
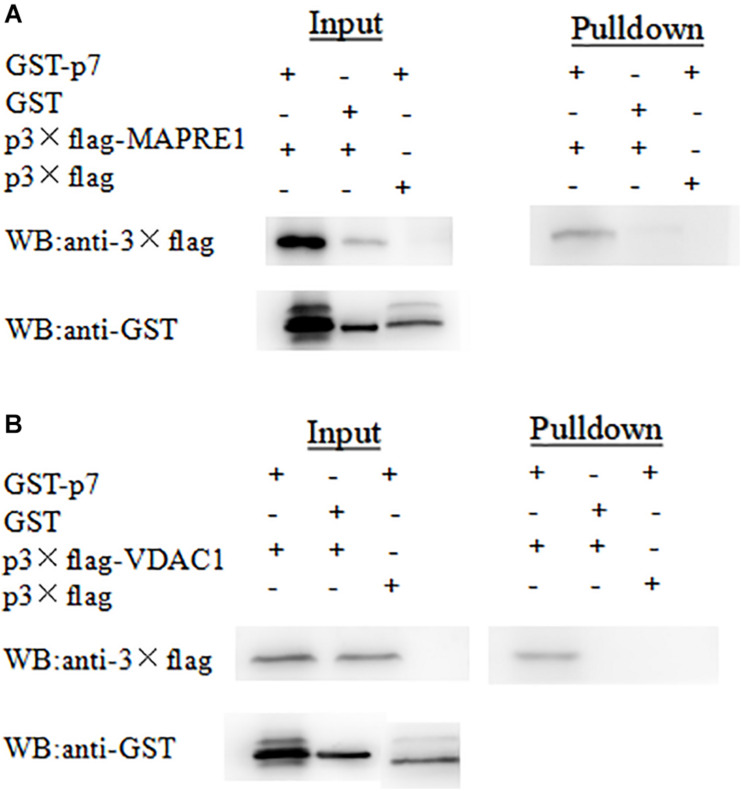
Identification of the interaction of CSFV p7 with MAPRE1 **(A)** and VDAC1 **(B)** by GST-pulldown assay, respectively. Glutathione beads conjugated to GST or the GST-p7 protein were incubated with recombinant Flag-MAPRE1 or Flag-VDAC1 protein. After washing, proteins were eluted from the beads and SDS-PAGE was performed. The expression of MAPRE1 or VDAC1 was determined by immunoblotting with anti-Flag mAb. GST and GST-p7 protein expression were confirmed by immunoblotting.

To further verify the interaction between p7 and the identified protein *in vitro*, a GST-pulldown assay was conducted with the GST-tagged p7 protein expressed in *E. coli* and p3 × Flag-tagged target protein expressed in HEK293T cells. Western blot results showed that GST-p7 could capture p3 × Flag-tagged MAPRE1 and VDAC1, respectively (Figure 3).

### Construction and Analysis of the p7-Cellular Protein Interaction Network

**FIGURE 4:**
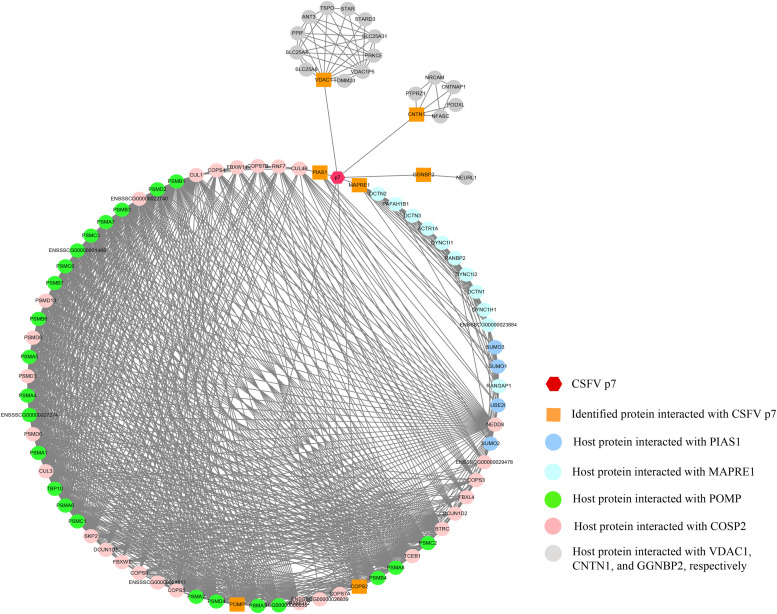
The network of interactions between CSFV p7 and host cellular proteins. The p7-cellular protein interaction network was constructed based on the interactions of proteins including CSFV p7, the identified proteins by Y2H screening, and other host proteins interacting with the identified proteins. In brief, the seven identified proteins screened by Y2H assay were inputted into the STRING database, while the specie was set to “*Sus scrofa*” and other parameters were the default settings. The total list of proteins directly and indirectly related to the seven identified proteins based on protein-protein interactions was obtained from the STRING database. Protein interactions from the STRING database with a combined score >0.7 were selected for network construction. Based on the experimental results screened by the Y2H system and the correlation between the proteins in the STRING database, the p7-cellular protein interaction network was built using the Cytoscape v3.7.2 software. In the network, the core protein p7 was marked as a hexagon. The orange squares indicated proteins identified by Y2H assay to interact with p7. The other proteins, which were identified as potentially interacting with the host proteins interacting with p7, were marked as circles. The interactions between proteins were expressed by straight lines between the nodes.

The p7-cellular protein interaction network was constructed based on the interactions of proteins including CSFV p7, the identified proteins, and other host proteins interacting with the identified proteins (Supplementary Table 1). It showed that 91 nodes representing CSFV p7 and 90 host proteins were connected via a total of 939 edges in the p7-cellular protein interaction network (Figure 4). In this network, POMP, COPS2, MAPRE1, and PIAS1 were the remarkable node proteins, whereas VDAC1, CNTN1, and GGNBP2 were sub-remarkable node proteins. The host proteins that interacted with POMP, COPS2, MAPRE1, and PIAS1 could form a circular interaction sub-network.

### GO and KEGG Enrichment of Host Proteins in the p7-Cellular Protein Interaction Network

**FIGURE 5:**
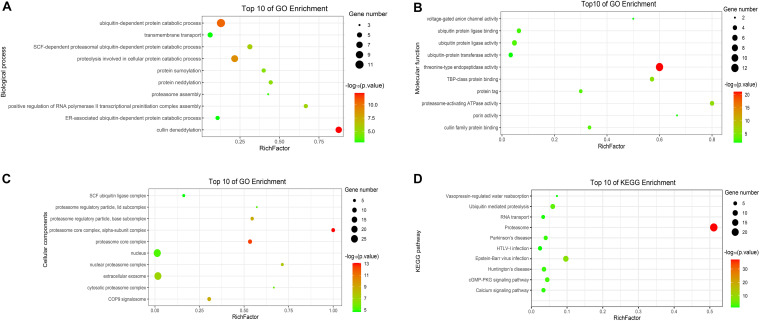
GO and KEGG enrichment of host cellular proteins in the p7-cellular protein interaction network. **(A)** Biological process of GO enrichment; **(B)** Molecular function of GO enrichment; **(C)** Cellular components of GO enrichment; **(D)** KEGG pathway enrichment. The abscissa represents the “RichFactor.” RichFactor = “count”/“pop hits.” The “count” is the number of hub genes enriched in a certain term. The “pop hits” is the number of all genes enriched in a certain term. The size of the bubbles indicates the “gene count.” The color of the bubbles indicates the “-log_10_(*p*-value).” A *p*-value < 0.05 is regarded as statistically significant for the correlations.

To analyze the functions and mechanisms of host proteins in the p7-cellular protein interaction network (Supplementary Table 2), GO annotation and analysis were performed for three categories including biological process, cellular component, and molecular function and then the top 10 most enriched GO terms according to *p*-value were shown in Figures 5A–C. The host proteins were enriched in cullin deneddylation, ubiquitin-dependent protein catabolic process, protein neddylation, protein sumoylation, proteasome assembly, and others according to the analysis of biological processes and were mainly found to be the component of the proteasome core complex, SCF ubiquitin ligase complex, extracellular exosome, and COP9 signalosome based on the analysis of cellular components. For molecular functions, the host proteins were mainly associated with threonine-type endopeptidase activity, proteasome-activating ATPase activity, ubiquitin-protein ligase activity, ubiquitin-protein transferase activity, cullin family protein binding, and others.

To further predict the cellular pathways of host proteins in the p7-cellular protein interaction network (Supplementary Table 2), KEGG pathway enrichment analysis was conducted and the top 10 enriched KEGG pathways according to *p*-value were shown in Figure 5D. Based on the results of the KEGG pathway analysis, we found that most of the host proteins were mainly enriched in the proteasome, ubiquitin-mediated proteolysis, cGMP-PKG signaling pathway, and calcium signaling pathway. Interestingly, the pathway of the proteasome was enriched with the lowest *p*-value and the highest number of genes.

## Discussion

Previous studies showed that several viruses could encode viroporin, such as the M2 of influenza virus (Rossman et al., 2010), the vpu protein of HIV (Gonzalez, 2015), the p7 of hepatitis C virus (Farag et al., 2017), and the 2B of poliovirus (Nieva et al., 2003). As viroporin could modulate the cellular ion balance and then take over the cellular machinery, blocking viroporin activity by specific inhibitors, or developing viroporin-defective attenuated vaccines offers new ways to treat or prevent viral infection (Nieva et al., 2012). Recently, CSFV p7 was confirmed to be a viroporin and was involved in CSFV virulence (Gladue et al., 2012), thus research on the CSFV p7 will be beneficial to the development of new vaccines or therapies for effectively controlling CSF.

**FIGURE 6:**
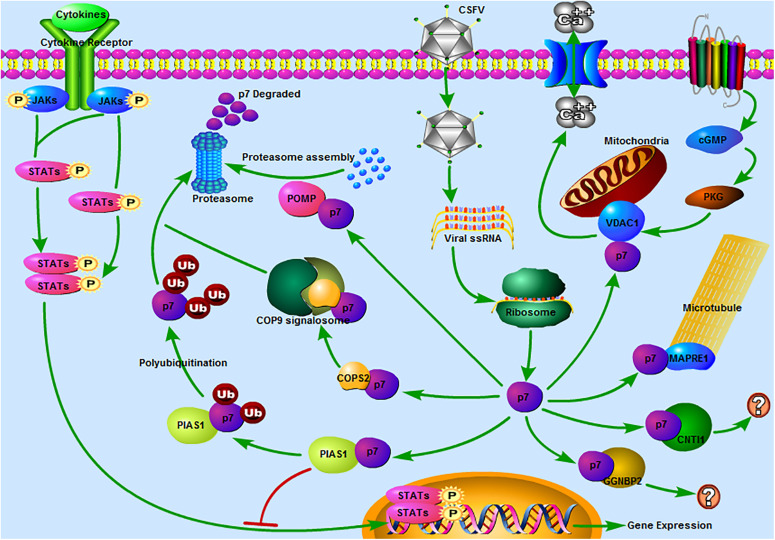
Predicted pathways might be involved in the interaction between CSFV p7 and host cells. 1 JAK-STAT pathway. Our previous study revealed that the JAK-STAT pathway might play a significant role in CSFV infection (Chen et al., 2020). In the present study, PIAS1 (protein inhibitor of activated STAT 1) was found to be a potential binding partner of the CSFV p7, which suggested that p7 might regulate the JAK-STAT pathway by interacting with PIAS1. 2 The ubiquitin-proteasome system (UPS). It was reported that p7 was a short-lived protein as it could be easily degraded by the proteasome (Lin et al., 2014). PIAS1 was involved in ubiquitin-mediated proteolysis based on the results of the KEGG pathway analysis. POMP (proteasome maturation protein) was related to the proteasome assembly. COPS2 (COP9 signalosome subunit 2) might be related to the UPS as the COP9 signalosome could regulate the UPS (Rao et al., 2020). Thus, PIAS1, POMP, and COPS2 might be involved in the UPS and CSFV p7 might have a close relationship with the UPS. 3 The calcium signaling pathway. The calcium signaling pathway was also enriched based on the KEGG pathway analysis and VDAC1 (voltage-dependent anion channel 1) was included in this pathway. Our previous work has demonstrated that the increase of [Ca^2+^]_*cyto*_ mediated by CSFV infection could potently induce autophagy (Xie et al., 2020). 4 The cGMP-PKG signaling pathway. The cGMP-PKG signaling pathway was predicted based on the KEGG pathway analysis and VDAC1 was included in this pathway. It was reported that the cGMP-PKG signaling pathway was associated with the replication of some viruses, such as spring viremia of carp virus (Li et al., 2018), porcine reproductive and respiratory syndrome virus (Zhang et al., 2017), and HIV-1 (Lee et al., 2007). Thus, we speculated that the cGMP-PKG signaling pathway might be related to CSFV replication. 5 Regulation of microtubule function. MAPRE1 (microtubule-associated protein RP/EB family member 1), was involved in protein localization to microtubule plus-end, regulation of microtubule polymerization or depolymerization. MAPRE1 was a potential binding partner of the p7, which suggested that p7 might be associated with the regulation of microtubule function. 6 Other pathways. CNTN1 (contactin 1) or GGNBP2 (gametogenetin binding protein 2) associated pathway was unclear. The predicted pathways were drawn using Pathway Builder Tool 2.0.

According to the functional analysis of proteins, it revealed that several potential binding partners of CSFV p7 were associated with the ubiquitin-proteasome system (UPS). Especially, PIAS1 was involved in ubiquitin-mediated proteolysis based on the results of KEGG pathway analysis, POMP (proteasome maturation protein) was related to the proteasome assembly, and COPS2 (COP9 signalosome subunit 2) might be related to the UPS as COP9 signalosome could regulate the UPS (Rao et al., 2020). We also found that many host proteins, such as CUL3, RNF7, BTRC, SKP2, UBE2I, TCEB1, PIAS1, CUL1, and different kinds of subunits of the proteasome, in the p7-cellular protein interaction network were related to the UPS, which suggested that CSFV p7 had a close relationship with the UPS (Figure 6). Some early studies also mirrored this speculation, given that they claimed that p7 was a short-lived protein that could be easily degraded by the proteasome (Lin et al., 2014). Our previous work also suggested that both capsid (C) protein and non-structural protein NS3 of CSFV could be degraded through the UPS (Chen et al., 2019; Deng et al., 2019). Besides, it has been proved that interactions of C protein with host cellular proteins SUMO-1 (small ubiquitin-like modifier) and UBC9 (a SUMO-1 conjugating enzyme) were pivotal for efficient viral proliferation and viral virulence (Gladue et al., 2010). Thus, we speculated that UPS might play a significant role in CSFV replication.

In addition to the UPS, other pathways were also enriched. We speculated that the calcium signaling pathway was involved in the interaction between p7 and host protein (Figure 6). This speculation was consistent with our previous work, which demonstrated that the increase of [Ca^2+^]_*cyto*_ mediated by CSFV infection could potently induce autophagy via the CAMKK2 (calcium/calmodulin-dependent protein kinase kinase 2) - PRKAA (protein kinase AMP-activated catalytic subunit alpha) - MTOR (mechanistic target of rapamycin kinase) pathway (Xie et al., 2020). Our previous study also showed that the JAK-STAT pathway might play a significant role in CSFV infection (Chen et al., 2020). In the present study, PIAS1, a protein inhibitor of activated STAT 1, was found to be a potential binding partner of the CSFV p7, implying that p7 might regulate the JAK-STAT pathway by interacting with PIAS1 (Figure 6). Furthermore, the cGMP-PKG signaling pathway was also worthy of attention (Figure 6). It was reported that the cGMP-PKG signaling pathway was associated with the replication of viruses (Lee et al., 2007; Zhang et al., 2017; Li et al., 2018). Carbon monoxide could inhibit the replication of porcine reproductive and respiratory syndrome virus by activating the cellular cGMP/PKG signaling pathway and negatively regulating cellular NF-κB signaling (Zhang et al., 2017). Heme Oxygenase-1 also inhibited the replication of spring viremia of carp virus through carbon monoxide mediated cGMP/PKG signaling pathway (Li et al., 2018). As the cGMP-PKG signaling pathway was associated with antiviral effect, it was valuable to study the relationship between the cGMP-PKG signaling pathway and CSFV infection, which has not been reported before. Additionally, host proteins were also involved in the pathways of Huntington’s disease, Parkinson’s disease, Epstein-Barr virus infection, and HTLV-I infection according to the KEGG analysis, which suggested that the research progress on these diseases could provide constructive information for the studies on CSF.

Combining the protein functional analysis in the current study with previous reports (Dumas et al., 2015; Jovasevic et al., 2015; Han et al., 2017), we also observed that MAPRE1 and VDAC1 were related to virus infection. MAPRE1, also known as end-binding protein 1 (EB1), acts as a master regulator of plus-end tracking proteins (+TIPs) networks by targeting the growing ends of microtubules and recruiting other + TIPs and finally regulates the microtubule dynamics (Galjart, 2010). Scholars found MAPRE1 was involved in virus infection (Dumas et al., 2015; Jovasevic et al., 2015). For example, the regulatory viral protein of HIV-1 could interact with MAPRE1, p150 (Glued), and dynein heavy chain and critically alter the microtubule plus end localization of MAPRE1 and p150 (Glued), hence altering the centripetal movement and maturation of phagosomes and impairing major functions of HIV-1 infected-macrophages (Dumas et al., 2015). Meanwhile, our findings indicated p7 could interact with MAPRE1. Together those findings hint that p7 might regulate the function of microtubules through interaction with MAPRE1.

Another interactive cellular protein, VDAC1, is located in the outer mitochondrial membrane, serves as a mitochondrial gatekeeper, and controls a wide variety of mitochondrial functions, such as controlling the metabolic and energy cross-talk between mitochondria and the rest of the cell, involvement in PINK1/Parkin-mediated mitophagy, participation in mitochondrial-mediated apoptosis and involvement in lipid metabolism and cholesterol transport (Geisler et al., 2010; Shoshan-Barmatz et al., 2010, 2015; Geula et al., 2012). VDAC1 was also involved in the infection of viruses, such as infectious bursal disease virus (IBDV). VDAC1 could interact with IBDV Ribonucleoprotein (RNPs) and facilitates IBDV replication by enhancing IBDV polymerase activity (Han et al., 2017). In our previous work, we have confirmed that CSFV infection was closely associated with mitochondrial function (Gou et al., 2017; Ma et al., 2019). CSFV infection could induce mitochondrial fission and mitophagy to inhibit host cell apoptosis for persistent infection (Gou et al., 2017). We also revealed that the process of free fatty acids entering the mitochondria for beta-oxidation to produce ATP was necessary for CSFV replication (Ma et al., 2019). Given that both the cGMP-PKG and the calcium signaling pathway were enriched based on the KEGG analysis and VDAC1 was a key molecule in these two pathways, it implied that p7 might regulate the cGMP-PKG and calcium signaling pathway through interaction with VDAC1. Moreover, the interaction of p7 with VDAC1 might also affect the activities of mitochondria, because VDAC1 was closely associated with mitochondrial function.

In addition to MAPRE1 and VDAC1, other host proteins identified by Y2H screening were also worthy of further study. We knew that the Y2H assay alone was not enough to confirm the interaction between CSFV p7 and host proteins. Thus, seven proteins screened by Y2H assay were considered as putative interacting proteins of CSFV p7. The p7-MAPRE1/VDAC1 interaction has been evaluated using Y2H, co-IP, and GST-pulldown assays in the present study. The p7-MAPRE1/VDAC1 co-localization in CSFV-infected cells will be further analyzed using laser confocal microscopy assay. Meanwhile, the other five candidate proteins interacting with p7 will be evaluated by other methods, including co-IP, GST-pulldown, and laser confocal microscopy assays in our next work. Particularly, the biological process related to the interaction of p7-CNTN1/GGNBP2 will be further explored, because there is less supporting evidence for effect of CNTN1 and GGNBP2 on viral infection in the literature. Besides, the involvement of interaction between p7 and the identified proteins in the CSFV life cycles and the effect of the identified proteins on CSFV infection will be clarified in the next work.

## Conclusion

In summary, our study identified seven potential interactive cellular proteins of CSFV p7 by utilizing the Y2H assay and the p7-cellular protein interaction network was constructed and analyzed. Based on the results of Y2H assay and bioinformatics analysis, we found that the ubiquitin-proteasome system might be involved in the interaction between CSFV p7 and host cells, consistent with previous observations that CSFV p7 was degraded by the proteasome. Besides, the JAK-STAT pathway, the calcium signaling pathway, the cGMP-PKG signaling pathway, and the regulation of microtubule function might be also involved in the interaction between CSFV p7 and host cells, which has not been reported before and was worthy of further study. This work is beneficial to reveal the role of p7 on the CSFV infection and also conducive to develop new vaccines or therapies for effectively controlling CSF.

## Data Availability Statement

The original contributions presented in the study are included in the article/Supplementary Material, further inquiries can be directed to the corresponding authors.

## Author Contributions

CJ, LY, HD, MZhao, and JC conceived and designed the study. JF, MZhu, ZZ, KW, ZL, WL, and SF performed the experiments. JF, MZhan, and CL carried out the data analysis and drafted the manuscript. All authors read and approved the final manuscript.

## Conflict of Interest

The authors declare that the research was conducted in the absence of any commercial or financial relationships that could be construed as a potential conflict of interest.

## Footnotes

1 http://string-db.org
